# Effects of antiviral therapy on post-hepatectomy HBV reactivation and liver function in HBV DNA-negative patients with HBV-related hepatocellular carcinoma

**DOI:** 10.18632/oncotarget.14789

**Published:** 2017-01-21

**Authors:** Wen-Feng Gong, Jian-Hong Zhong, Shi-Dong Lu, Xiao-Bo Wang, Qiu-Ming Zhang, Liang Ma, Zhi-Ming Zhang, Bang-De Xiang, Le-Qun Li

**Affiliations:** ^1^ Hepatobiliary Surgery Department, Affiliated Tumor Hospital of Guangxi Medical University, Nanning 530021, China; ^2^ Guangxi Liver Cancer Diagnosis and Treatment Engineering and Technology Research Center, Nanning 530021, China; ^3^ General Medicine Department, The First People Hospital of Qinzhou, Qinzhou, 535000, China

**Keywords:** hepatocellular carcinoma, hepatitis B virus, antiviral, reactivation, liver function

## Abstract

The ability of antiviral therapy to reduce risk of post-hepatectomy hepatitis B virus (HBV) reactivation in patients negative for viral DNA is unclear. This prospective study involved 174 consecutive patients with hepatitis B virus related hepatocellular carcinoma who were negative for hepatitis B virus DNA in serum and who underwent hepatic resection. Hepatitis B virus reactivation occurred in 30 patients in the non-antiviral group (27.8%) but in only 2 patients in the antiviral group (3.0%, *P* < 0.001). Based on multivariate analysis, risk of hepatitis B virus reactivation was associated with minor hepatectomy and absence of antiviral therapy. Liver function indicators at one week after resection did not differ significantly between the two groups, or between patients who experienced hepatitis B virus reactivation or not. Nevertheless, alanine aminotransferase and albumin at 1 month after resection were significantly higher in the antiviral group than in the non-antiviral group, and they were significantly higher in patients who did not experience hepatitis B virus reactivation than in those who did. Therefore, patients with hepatitis B virus related hepatocellular carcinoma face substantial risk of hepatitis B virus reactivation after hepatectomy, even if they are negative for viral DNA at baseline. Antiviral therapy can reduce the risk of reactivation, helping improve liver function after surgery. (Clinicaltrials.gov registration number: NCT02829359).

## INTRODUCTION

Hepatocellular carcinoma (HCC) is the fifth most common cancer worldwide and the third most common cause of cancer mortality [1, 3]. In Asian countries, hepatitis B virus (HBV) infection has been identified as an important etiological factor in hepatocarcinogenesis. Patients with HBV-related HCC therefore require pre- or postoperative antiviral treatment, which can induce remission of active hepatitis, help maintain hepatic function, prevent HCC recurrence and increase the likelihood of successful treatment if the disease does recur [4].

Such postoperative antiviral therapy may not be enough. Numerous studies have shown that conformal radiotherapy [5–6], systemic chemotherapy [7–8], radiofrequency ablation [9] and transcatheter arterial chemoembolization (TACE) [10–11] can lead to HBV reactivation and hepatic dysfunction during treatment. Hepatectomy of patients with HBV-related HCC who are positive for HBV DNA in serum can lead to HBV reactivation during the perioperative period, and patients positive for HBV DNA or HBV surface antigen (HBsAg) may be at particularly high risk of reactivation [7, 9]. HBV reactivation can degrade liver function [12], aggravateliver cirrhosis [13], increase the risk of HBV-related HCC recurrence [14], and increase the incidence and severity of potentially life-threatening complications [15]. For these reasons, official guidelines [16–19] recommend simultaneous comprehensive anti-tumor treatment and antiviral treatment for HCC patients positive for HBV DNA.

It is unclear whether antiviral therapy during the perioperative period can also benefit patients who have HBV-related HCC but who are *negative* for HBV DNA. The present study evaluated the incidence of post-hepatectomy HBV reactivation in such patients and explored the risk factors of reactivation. The influence of HBV reactivation on liver function recovery was also analyzed.

## RESULTS

### Study population characteristics

**Figure 1:**
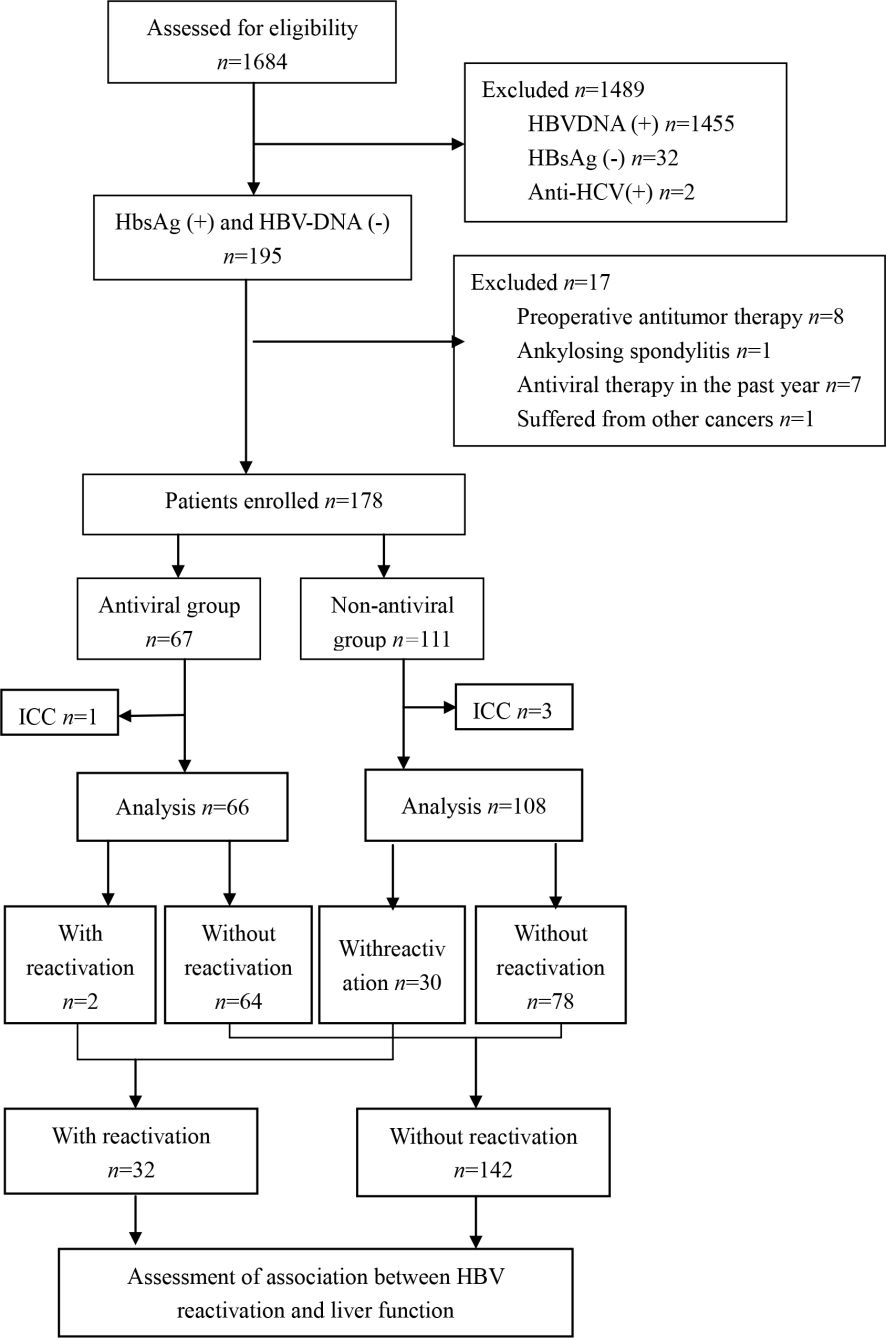
Patient enrollment and assessment

During the enrollment period, 1,684 potentially eligible patients were admitted for initial hepatectomy at our hospital between July 2012 and June 2016. Of these, 1489 were excluded because they were negative for serum HBsAg (*n* = 32), positive for HBV DNA (*n* = 1455) or positive for antibodies against hepatitis C virus (*n* = 2). A total of 195 patients who had HBV-related HCC and who were positive for serum HBsAg and negative for HBV DNA prior to hepatectomy were enrolled. After enrollment, 17 patients were excluded because they received TACE or other antitumor therapy before hepatectomy (*n* = 8), they had previously received antiviral therapy within one year of study enrollment (*n* = 7), or they had another cancer (*n* = 1) or autoimmune disease (*n* = 1). Another 4 patients were excluded when postoperative histopathology showed them to have intrahepatic cholangiocarcinoma. In the end, 66 patients were included in the antiviral group and 108 in the non-antiviral group (Figure 1).

The proportion of patients receiving minor hepatectomy was significantly higher in the antiviral group (*P* = 0.041), who also had a significantly lower mean albumin level than the non-antiviral group (*P* = 0.032). The two groups were similar across all other variables analyzed (all *P* > 0.05; Table 1).

**Table 1:** Baseline characteristics of included patients

Variable	Antiviral group(*n* = 66)	Non-antiviral group(*n* = 108)	*P*
Sex (M/F)	58/8	92/16	0.617
Age (yr)	49.97 ± 9.71	49.67 ± 12.22	0.904
BCLC stage (A/B/C)	42/12/12	60/20/28	0.466
Tumor number (< 3/≥ 3)	56/10	98/10	0.237
Tumor size (> 5/≤ 5 cm)	34/32	62/46	0.448
Preoperative tumor rupture (yes/no)	4/62	6/102	1.000
Blood loss (mL)	448.42 ± 303.73	459.26 ± 219.74	0.848
Blood transfusion (yes/no)	2/64	8/100	0.322
Operative time (min)	202.58 ± 69.41	195.50 ± 53.65	0.595
Tumor capsule (complete/absent + incomplete)	44/22	64/44	0.329
Anatomical hepatectomy (yes/no)	12/54	34/74	0.054
Inflow blood occlusion time (min)	28.03 ± 25.40	24.98 ± 38.88	0.690
Liver cirrhosis (present/absent)	54/12	87/21	0.837
Alpha fetoprotein ≥ 400 ng/mL	32	50	0.779
Prothrombin time (s)	12.82 ± 1.29	12.77 ± 1.41	0.857
Total bilirubin (μmol/L)	10.50 ± 3.85	20.17 ± 5.37	0.303
Albumin (g/L)	40.24 ± 5.19	42.57 ± 4.61	0.032
Alanine aminotransferase (IU/L)	20.97 ± 15.08	22.39 ± 17.61	0.617
Ascites (present/absent)	2/64	2/106	0.635
Types of hepatectomy (major/minor)	4/62	18/90	0.041
Hepatitis B virus Pre-S1 antigen (positive/negative)	46/20	60/48	0.064

BCLC, Barcelona Clinic Liver Cancer.

### HBV reactivation

By 1-month follow-up, HBV reactivation had occurred in a significantly larger proportion of patients in the non-antiviral group [30/108, (27.8%)] than in the antiviral group [2/64 (3.0%), *P* < 0.001]. Most reactivation events occurred soon after surgery: 8 patients experienced initial reactivation on postoperative day 1, 16 patients on day 3 (including the two patients in the antiviral group), 5 patients on day 5, 2 patients on day 7, and 1 patient on day 9. Patients who experienced reactivation were considered for antiviral therapy (Table 2).

**Table 2:** HBV DNA levels in patients in the antiviral and non-antiviral groups who experienced HBV reactivation within 1 month of hepatectomy

Day Patient	D0	D1	D3	D5	D7	D9	D30
1	≥	872	826	893	840	795	780
2	≥	500	771	795	780	764	750
3	≥	1012	1025	1109	1018	997	1007
4	≥	695	708	713	677	701	700
5	≥	598	631	621	587	600	624
6	≥	725	713	751	720	704	700
7	≥	818	843	819	800	792	805
8	≥	796	826	819	804	788	827
9	≥	≥	912	897	901	850	829
10	≥	≥	738	725	771	698	708
11	≥	≥	852	842	880	799	813
12	≥	≥	924	854	869	941	850
13	≥	≥	713	402	750	692	700
14	≥	≥	816	800	806	794	750
15	≥	≥	903	885	897	904	916
16	≥	≥	759	746	782	729	741
17	≥	≥	685	653	674	627	608
18	≥	≥	599	586	607	581	620
19	≥	≥	619	627	641	608	574
20	≥	≥	598	571	588	559	607
21	≥	≥	667	695	684	673	650
22	≥	≥	652	677	692	628	672
23	≥	≥	954	928	973	900	929
24	≥	≥	856	849	872	827	867
25	≥	≥	≥	873	859	894	807
26	≥	≥	≥	691	685	726	708
27	≥	≥	≥	655	639	675	649
28	≥	≥	≥	863	855	849	895
29	≥	≥	≥	529	537	542	518
30	≥	≥	≥	≥	639	622	640
31*	≥	≥	≥	≥	593	607	612
32*	≥	≥	≥	≥	≥	526	538

* Patients in the antiviral group who experienced HBV reactivation. D0, preoperative HBV DNA level (baseline); D1, D3, D5, D7, D9, D30 etc., postoperative HBV DNA levels; ≥, HBV DNA level < 500 IU/ml.

Univariate analysis identified the following risk factors of perioperative HBV reactivation (all *P* < 0.05; Table 3): cirrhosis, low serum albumin, minor hepatectomy and absence of antiviral therapy. Multivariate analysis identified only two risk factors (*P* < 0.05; Table 4): minor hepatectomy and absence of antiviral therapy.

**Table 3:** Univariate analysis to identify factors related to perioperative HBV reactivation

Variable	HBV reactivation (*n* = 32)	No HBV reactivation (*n* = 142)	*P*
Sex (M/F)	28/4	122/20	1.000
Age (yr)	50.25 ± 11.01	49.68 ± 11.41	0.855
BCLC-stage (A/B/C)	16/10/6	86/22/34	0.115
Tumor numbers (< 3/≥ 3)	28/4	126/16	0.766
Tumor size (> 5/≤ 5cm)	16/16	80/62	0.515
Tumor size(cm)	5.83 ± 2.60	6.94 ± 4.08	0.299
Preoperative tumor rupture (yes/no)	2/30	8/134	1.000
Blood loss (mL)	323.13 ± 238.38	362.37 ± 257.49	0.578
Blood transfusion (yes/no)	3/29	7/135	0.394
Operative time(min)	194.38±53.26	199.04±61.52	0.780
Tumor capsule (complete/ absent+ incomplete)	20/12	88/54	0.956
Anatomical hepatectomy (yes/no)	8/24	38/104	0.838
Inflow blood occlusion time (min)	21.00 ± 14.72	25.04 ± 23.61	0.514
Liver cirrhosis (present/absent)	30/2	111/31	0.042
Alpha fetoprotein ≥ 400 ng/mL	12	70	0.227
Prothrombin time (s)	12.95 ± 1.95	12.75 ± 1.20	0.599
Total bilirubin (μmol/L)	13.98 ± 6.43	17.08 ± 46.70	0.792
Albumin (g/L)	44.68 ± 2.12	41.01 ± 5.15	0.007
Alanine aminotransferase (IU/L)	34.38 ± 14.35	33.27 ± 23.16	0.855
Ascites (present/absent)	1/31	3/139	0.560
Types of hepatectomy (major/minor)	24/8	128/14	0.020
Hepatitis B virus Pre-S1 antigen (positive/negative)	20/12	86/56	0.839
Antiviral therapy (yes/no)	2/30	64/78	< 0.001

BCLC, Barcelona Clinic Liver Cancer.

**Table 4:** Multivariate analysis with logistic regression to identify factors related to perioperative HBV reactivation

Variable	Hazard ratio	95% confidence interval	*P*
Liver cirrhosis (present)	5.804	0.888–37.945	0.066
Albumin ≤ 35g/L	0.452	0.045–4.569	0.501
Minor hepatectomy (≤ 3 liver segments)	4.695	1.257–17.537	0.021
Noantiviral therapy	8.164	1.831–36.397	0.006

### Effect of HBV reactivation on recovery of liver function

**Figure 2:**
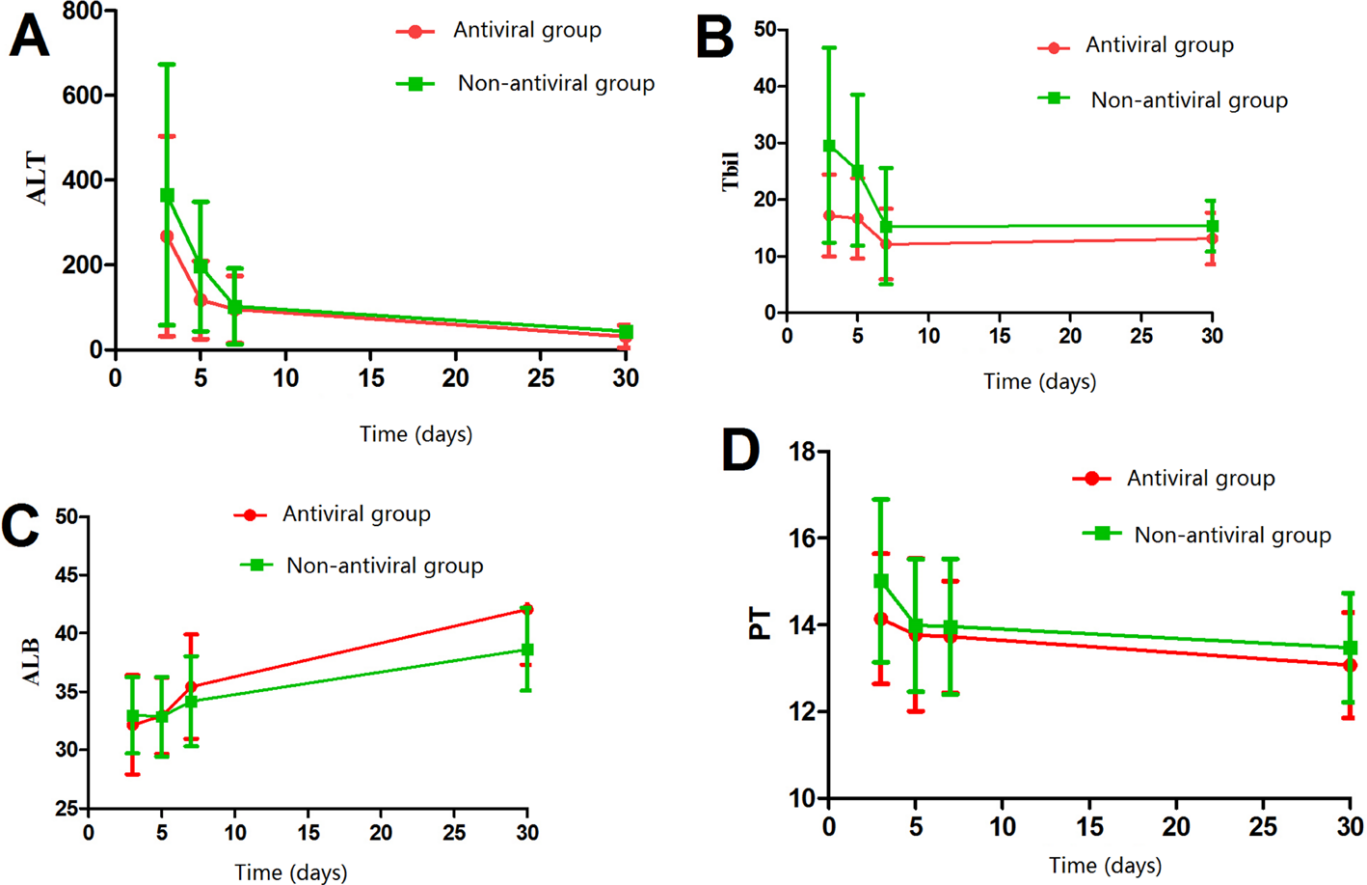
Comparison of liver function between patients with or without perioperative antiviral therapy

**Figure 3:**
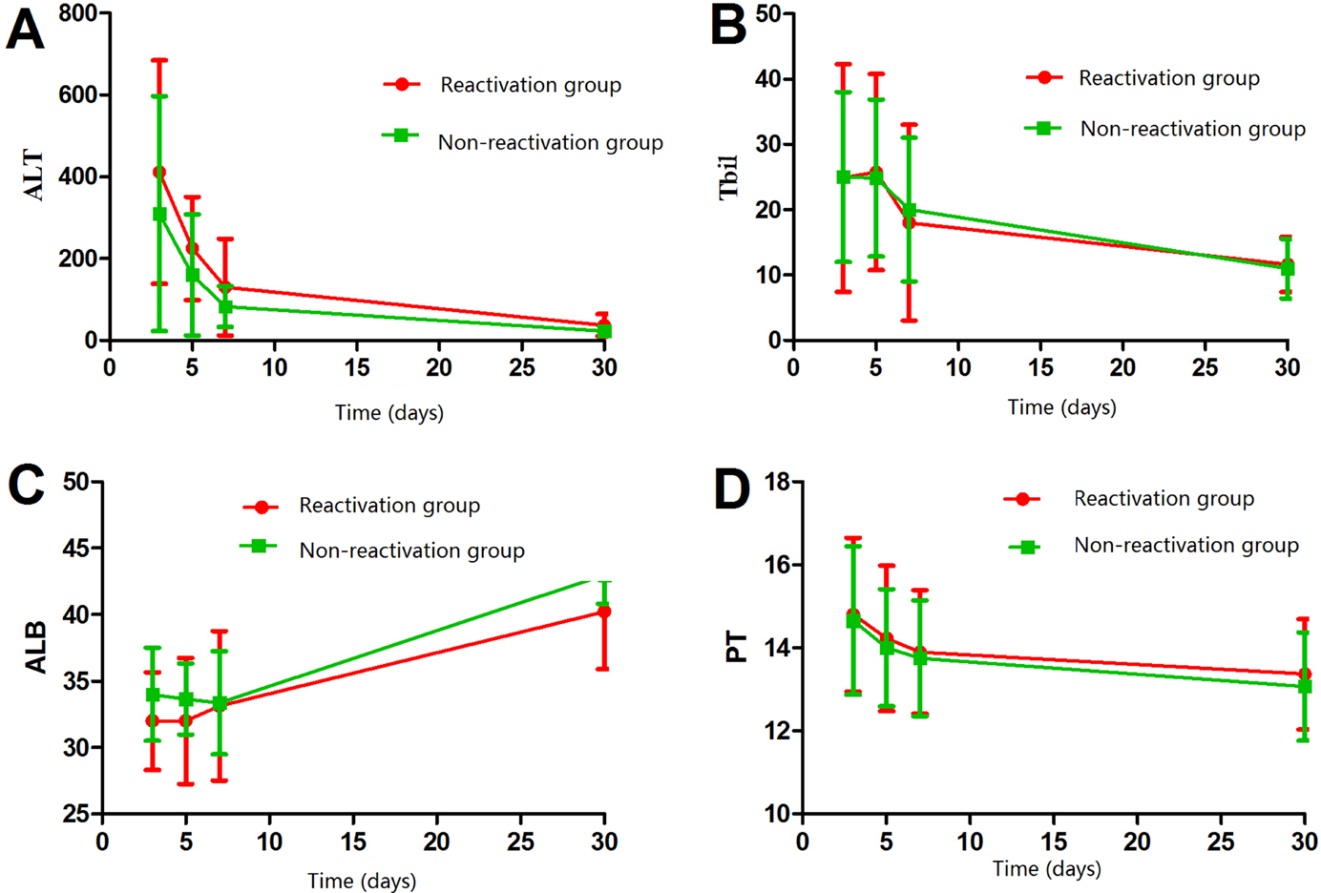
Comparison of liver function between patients who experienced post-hepatectomy HBV reactivation or not

At 1 week after hepatectomy, the antiviral and non-antiviral groups showed similar liver function in terms of alanine aminotransferase, total bilirubin, albumin, and prothrombin time (all *P* > 0.05; Figure 2). However, alanine aminotransferase was significantly lower and albumin significantly higher in the antiviral group than in the non-antiviral group at 1 month after hepatectomy, indicating better liver function (both *P* < 0.05; Figure 2). Stratifying patients according to whether they experienced HBV reactivation or not revealed no significant differences in alanine aminotransferase, total bilirubin, albumin, or prothrombin time at 1 week after surgery. At 1 month after hepatectomy, however, liver function was better in patients without HBV reactivation than in those who experienced it (Figure 3).

## DISCUSSION

The major drug treatments against chronic HBV infection, interferon and nucleoside/nucleotide analogues, usually do not completely eliminate the virus [20]. Even with these treatments, some copies of HBV covalently closed circular DNA persist in infected hepatocytes [21]. As a result, immunosuppressive drug therapy and surgery can trigger genome amplification, leading in turn to HBV reactivation [19]. For patients with HBV-related HCC who are positive for HBV DNA, official guidelines recommend entecavir and tenofovir as first-line postoperative antiviral therapy to suppress viral replication and thereby help improve liver function, decrease tumor recurrence, and prolong overall survival [16–18]. The present study shows that perioperative antiviral therapy with entecavir can also improve prognosis of patients who are negative for HBV DNA.

To our knowledge, this is the first systematic study to explore the incidence of HBV reactivation after hepatectomy in HCC patients who are positive for serum HBsAg but negative for HBV DNA at baseline. Our results show a 9-fold reduction in the rate of HBV reactivation within 1 month of hepatectomy when antiviral therapy is begun a few days before surgery and continued for at least 1 month afterwards.

Our results indicate that HBV reactivation is a serious concern even for HCC patients who are negative for viral DNA at baseline. This extends and complements an already extensive literature documenting the substantial risk of reactivation in patients positive for viral DNA. In one study of 55 HCC patients with high levels of HBV DNA (≥ 7 mEq/ml) at baseline, post-hepatectomy reactivation occurred in 28% and hepatitis in 24% [22]. Another study of HCC patients with high HBV DNA levels (≥ 2.0 × 10^3^ IU/ml) reported rates of HBV reactivation during the perioperative period of 31.8% (14/44) in the non-antiviral group and 2.5% (1/40) in the antiviral group [23]. HBV reactivation rates also appear to be relatively high for patients with lower levels of viral DNA. In a study of HCC patients with preoperative HBV DNA levels < 2.0 × 10^2^ IU/ml, reactivation occurred after hepatectomy in 21.1% without antiviral therapy [7]. In a study of patients with preoperative HBV DNA < 2000 IU/ml, reactivation occurred after resection in 19.1% [12]. This last study identified HBV DNA levels > 200 IU/ml and HBV reactivation as independent risk factors of poor disease-free and overall survival. The present results with HBV DNA-negative HCC patients, together with the existing literature on DNA-positive patients, strongly suggest that all HBsAg-positive HCC patients face substantial risk of postoperative reactivation. Thus, it may be beneficial to routinely administer prophylactic antiviral treatment to such patients before and immediately after hepatectomy.

How HBV reactivation occurs remains unclear. Hepatectomy may induce immunosuppression and thereby lead to HBV replication [24–26]. Subsequent recovery of immune function results in rapid destruction of infected hepatocytes, causing postoperative hepatitis and acute hepatic failure. In addition, HBV can integrate into liver cells and persist for long periods, protected from immune monitoring. As liver cells regenerate following hepatectomy, HBV can infect these cells because of its strong hepatotropism, causing viral load to increase.

In our cohort, minor hepatectomy was a risk factor of HBV reactivation. How this procedure can reactivate viral replication is poorly understood. We attribute this association to the issue of residual viral load. Major hepatectomy, in theory, eliminates a large amount of HBV load, and reactivation in the remnant liver should be difficult to detect since relatively little hepatic tissue remains and the cells regenerate slowly. In minor hepatectomy, however, remnant liver volume is large and so is the residual viral load. The residual liver tissue can regenerate fairly quickly, and the abundant HBV can infect the new tissue relatively easily. Thus, it stands to reason that minor hepatectomy leads to greater risk of postoperative HBV reactivation than the corresponding major procedure. We did not observe that liver cirrhosis, albumin ≤ 35 g/L or other clinical factors correlated significantly with HBV reactivation after hepatectomy.

Our results suggest that although the use of prophylactic antiviral therapy and occurrence of HBV reactivation do not appear to significantly improve liver function within 1 week after hepatectomy, they can influence function at 1 month. This may reflect the fact that liver function soon after hepatectomy reflects primarily damage due to the surgery itself, whereas damage over longer periods reflects the continuous processes that give rise to reactivation. Our results are consistent with previous reports that antiviral therapy can reduce HBV reactivation-induced liver damage, improve liver function, promote hepatocyte regeneration and increase the volume of residual liver after hepatectomy [27–29]. Since our study focused on the perioperative period, we did not conduct longer follow-up. Future studies should be repeated with longer follow-up to examine the prognosis of HBV DNA-negative patients with HBV-related HCC who receive prophylactic antiviral therapy.

In addition to its short follow-up, our study has several other limitations. During the first week, we did not assay liver function using the ascites test, even though this is one of the indices for Child-Pugh assessment of liver function. We did not use this test because the presence of ascites need not indicate poor liver function; it can also occur as a result of inflammation, lymphatic blockage, and changes in plasma osmotic pressure [30]. In addition, our study was based on a small sample, and our detection limit of 500 IU/ml of HBV DNA may not be sufficiently sensitive to detect low levels of viral replication. We did not genotype the HBV strains in our patients, leaving open the question of whether genotype influences risk of HBV reactivation. Last, patients were not randomized allocated. This study has the inherent defect of comparing management and surgery by different teams of surgeons, thus introducing possible biases into this study.

While our cohort contained only patients undergoing initial hepatectomy, HCC recurs in up to 75% of patients by 5 years after initial surgery [31]. Such patients must then undergo TACE, radiofrequency ablation, anhydrous alcohol injection, three-dimensional conformal radiotherapy, some other comprehensive multimodal treatment or even repeat surgery [32–33]. All these treatments can trigger HBV reactivation and further compromise liver function. Therefore our work demonstrating the clinical benefit of prophylactic antiviral therapy is likely to be relevant for patients with initial and recurrent HCC. In particular, our results support the use of perioperative antiviral therapy in HCC patients who are positive for HBsAg and negative for HBV DNA. Our results should be confirmed and extended in larger studies with longer follow-up.

## MATERIALS AND METHODS

### Consent and ethical approval statement

Written informed consent was obtained from the patients in this study prior to their undergoing hepatectomy. All procedures in this study were in accordance with the rules of the Research Ethics Committee of the Affiliated Tumor Hospital of Guangxi Medical University and with the 1964 Helsinki Declaration and its amendments.

### Patients

This prospective study involved 174 consecutive patients with HBV-related HCC who underwent hepatectomy between July 2012 and June 2016 at the Affiliated Tumor Hospital of Guangxi Medical University. All patients were assayed for HBsAg and its antibody, hepatitis B e antigen and its antibody, antibody to HBV core antigen, HBV Pre-S1, antibody to hepatitis C virus, HBV DNA, serum alanine aminotransferase, serum albumin, prealbumin, total bilirubin, prothrombin time, and alpha-fetoprotein. Patients also underwent computed tomography of the liver one week before surgery. The trial is retrospectively registered (July 11, 2016) at Clinicaltrials.gov (NCT02829359).

Patients were included in the present study if they satisfied all the following inclusion criteria: (a) they underwent initial hepatectomy, (b) they were positive for serum HBsAg before surgery, (c) they were negative for serum HBV DNA (< 500 IU/ml); (d) their serum alanine aminotransferase was within the normal range, (e) they had Child-Pugh A liver function, (f) their HCC was confirmed by histopathology, and (g) they received either entecavir antiviral therapy or no antiviral therapy before (not exceeding 7 days) and after hepatectomy.

Patients were excluded from this trial if they met any of the following criteria: (a) they received TACE or other anti-tumor treatments prior to hepatectomy; (b) they had previously received antiviral treatment within 12 months of entering the study; or (c) they had autoimmune disease, malignant tumors in other organs, or other severe disease.

Levels of HBV DNA in serum were quantified using real-time polymerase chain reaction coupled to a fluorescence assay in a commercial hepatitis detection kit (DaAn Gene, Guangzhou, China). The manufacturer-specified lower limit of quantification was 500 IU/ml. HBV reactivation was defined as a shift in HBV DNA levels from being undetectable at baseline to being detectable. Liver function was compared before and after hepatectomy using the indices alanine aminotransferase, total bilirubin, albumin, and prothrombin time.

### Patient allocation and perioperative management

Our two Hepatobiliary Surgery Departments are divided into several medical teams by the senior doctors. In three medical teams, patients who met the inclusion criteria would receive entecavir (0.5 mg/d; Zhengda Tianqing, Lianyungang, China) starting 3 days before hepatectomy and for at least 1 month afterwards. These patients were assigned as the antiviral group. In the other medical teams, patients did not receive any antiviral therapy and they would be assigned as the non-antiviral group. Patients in the non-antiviral group who experienced HBV reactivation were considered for entecavir therapy after enrollment in the study, as per standard practices [16]. None of the patients in our study received immunological therapy during the perioperative period (defined as during hepatectomy or up to 1 month afterwards).

Hepatectomy was performed as described [34–35]. Perioperative data were recorded about type of hepatectomy, blood loss volume, tumor size, tumor capsule, duration of porta hepatis clamping, and duration of surgery. Data were also recorded about transfusion of red cells and blood plasma and about infusion of human blood albumin during and after hepatectomy. Indications for transfusions and infusions were as described [36–37]. The duration of inflow blood occlusion was defined to be the duration of porta hepatis clamping. Major hepatectomy was defined as the resection of three or more Couinaud segments; minor hepactectomy, as the resection of fewer than three segments.

On postoperative days 1–7, all patients received drug therapy [Magnesium Isoglycyrrhizinate for Injection, 150 mg/d, Zhengda Tianqing, Lianyungang, China; Ademetionine 1,4-Butanedisulfonate for Injection, 1000mg/d, Abbott S.P.A, Via Pontina Km 5204010 Campoverde (Aprilia) LT, Italy] to protect liver function and lower transaminase levels. HBVDNA levels, liver function, and blood clotting function were assayed on days 1, 3, 5, 7, 9 and 30 after hepatectomy. Criteria for hospital discharge included stability of vital signs with no fever, ability to tolerate solid food without vomiting, absence of other postoperative complications, control of postoperative pain, and ability to function at home independently or with home care [38].

### Statistical analysis

Continuous data were reported as mean and standard deviation and compared between patient groups using the independent-samples *t* test. Categorical data were reported as percentages and compared between patient groups using the chi-squared or Fisher's exact tests as appropriate. Multivariate analysis with logistic regression was used to identify risk factors of HBV reactivation. The threshold of significance was defined as two-tailed *P* < 0.05. Data were analyzed using SPSS 17.0 (IBM, USA).

## CONCLUSIONS

We provide evidence that liver resection can trigger HBV reactivation during the perioperative period in patients who have HBV-related HCC and who are negative for serum HBV DNA. Our results further suggest that the risk of reactivation is significantly lower when such patients are given perioperative prophylactic antiviral therapy. Reducing the risk of reactivation helps improve postoperative liver function. However, the long-term efficacy of entecavir for such patients was unknown. Besides, patient without liver cirrhosis was not analyzed specifically. Therefore, our conclusions remain highly preliminary and must be verified in much larger studies with longer follow-up that take into account these limitations.
